# UAV telephotography elucidates floristic variability and beta diversity of island cliffs under grazing interventions

**DOI:** 10.1038/s41598-024-66446-7

**Published:** 2024-07-05

**Authors:** Seongjun Kim, Chang Woo Lee, Hwan-Joon Park, Jung Eun Hwang, Hyeong Bin Park, Young-Jun Yoon, Yeong-Joong Kim

**Affiliations:** Research Center for Endangered Species, National Institute of Ecology, Yeongyang, Gyeongbuk Province 36531 Republic of Korea

**Keywords:** Alien species, Drone, Introduced herbivore, Plant species composition, Unreachable cliff, Biodiversity, Plant ecology, Community ecology

## Abstract

Cliffs contain one of the least known plant communities, which has been overlooked in biodiversity assessments due to the inherent inaccessibility. Our study adopted the unmanned aerial vehicle (UAV) with the telephoto camera to remotely clarify floristic variability across unreachable cliffs. Studied cliffs comprised 17 coastal and 13 inland cliffs in Gageodo of South Korea, among which 9 and 5 cliffs were grazed by the introduced cliff-dwelling goats. The UAV telephotography showed 154 and 166 plant species from coastal and inland cliffs, respectively. Inland cliffs contained more vascular plant species (*P* < 0.001), increased proportions of fern and woody species (*P* < 0.05), and decreased proportion of herbaceous species (*P* < 0.001) than coastal cliffs. It was also found that coastal and inland cliffs differed in the species composition (*P* < 0.001) rather than taxonomic beta diversity (*P* = 0.29). Furthermore, grazed coastal cliffs featured the elevated proportions of alien and annual herb species than ungrazed coastal cliffs (*P* < 0.05). This suggests that coastal cliffs might not be totally immune to grazing if the introduced herbivores are able to access cliff microhabitats; therefore, such anthropogenic introduction of cliff-dwelling herbivores should be excluded to conserve the native cliff plant communities.

## Introduction

Cliff is one of the valuable ecosystems in terms of its unique topography and ecological integrity^1^. Inherent inaccessibility of cliffs has been traditionally considered to provide refuges for many species vulnerable to competitions and land use changes^2–4^. Environmental gradients along the vertical cliff landscape result in heterogenous taxonomic and lifeform compositions and high biodiversity within a relatively small area^5–7^. Recently, plant ecologists further emphasize the necessity of studying cliff flora, given morphological differences from the non-cliff flora^8^, specialized interactions with the surrounding fauna and microclimate^9,10^, biological invasions of alien species^11^, and impacts from leisure climbing and grazing of introduced ungulates^12,13^.

Cliff plants are among the least known plant communities, which has been overlooked in biodiversity assessments due to the poor accessibility^14^. Most of the cliff studies have depended on the engagements of climbing and rappelling experts, which requires risky, time-consuming, and labor-intensive works^15^. Such direct investigations are also affected by the bias to the limited sampling points on accessible and visible cliffs, and consequently cause the knowledge gap regarding unreachable cliff habitats^16–19^. Moreover, the use of climbing tools during the direct investigations may disturb cliff microtopography, and hinder the health and structure of the studied plant communities^20,21^.

Rapid developments of unmanned aerial vehicle (UAV) act as a remote sensing platform to revolutionize monitoring methodologies for unreachable cliff ecosystems^22^. First of all, smart-controlled positioning systems enabled semi-automated flight and image sampling along the extremely steep landscapes^23^. The UAV studies have also collaborated with 3D modeling^24^, AI technology^25^, GIS database^26^, and robotics^27–29^ to enlarge the applicability and usefulness of UAV remote sensing. In addition, preliminary field trials with a close-range photography provided a guideline for the UAV-based image sampling on the harsh, vertical ecosystems^8,30^. The attachment of high-quality gimbal and telephoto lens can further improve such close-range UAV photography by allowing more detailed species identification from the safer distance, although it was limitedly piloted for insect ecology^31^.

Despite these innovations, UAV-based researches on cliff plant communities are scarce because the majority of UAV applications have focused on technical aspects of remote sensing and data analysis rather than hypothesis-driven ecological studies^22^; correspondingly, only few UAV studies targeted on the structure and function of cliff plant communities. For example, Zhou et al.^8^ reported an elevating number of plant species with increasing cliff height and decreasing distance from stream across subtropical inland cliffs. Kim et al.^30^ found that north-facing cliffs contained fewer evergreen species and more deciduous species than south-facing cliffs along temperate island coasts. Other studies surveyed the microhabitat characteristics of endangered cliff-dwelling plants^23,26^. However, such UAV studies principally addressed the counts of detected individuals and species but did not adopt the beta diversity index, which has become a key concept to understand ecological processes in relation to various natural and anthropogenic factors^32^.

Our study used UAV telephotography to remotely unveil the structure and beta diversity of plant communities along unreachable cliff landscapes. An island under grazing of introduced goats was selected to specifically compare floristic composition and beta diversity depending on cliff topography and cliff-dwelling herbivores. Given the previous reports on the effects of cliff plant communities^8,13,30,32^, we set two null hypotheses as follows: (1) cliffs directly exposed to the ocean surface (coastal cliffs) would differ in the flora and beta diversity from cliffs sheltered by vegetated, terrestrial landscapes (inland cliffs) and (2) grazing of introduced cliff-dwelling herbivores would reorganize the species composition and beta diversity by accelerating biological invasions into the native cliff plant communities.

## Results

### Flora taxa

**Figure 1:**
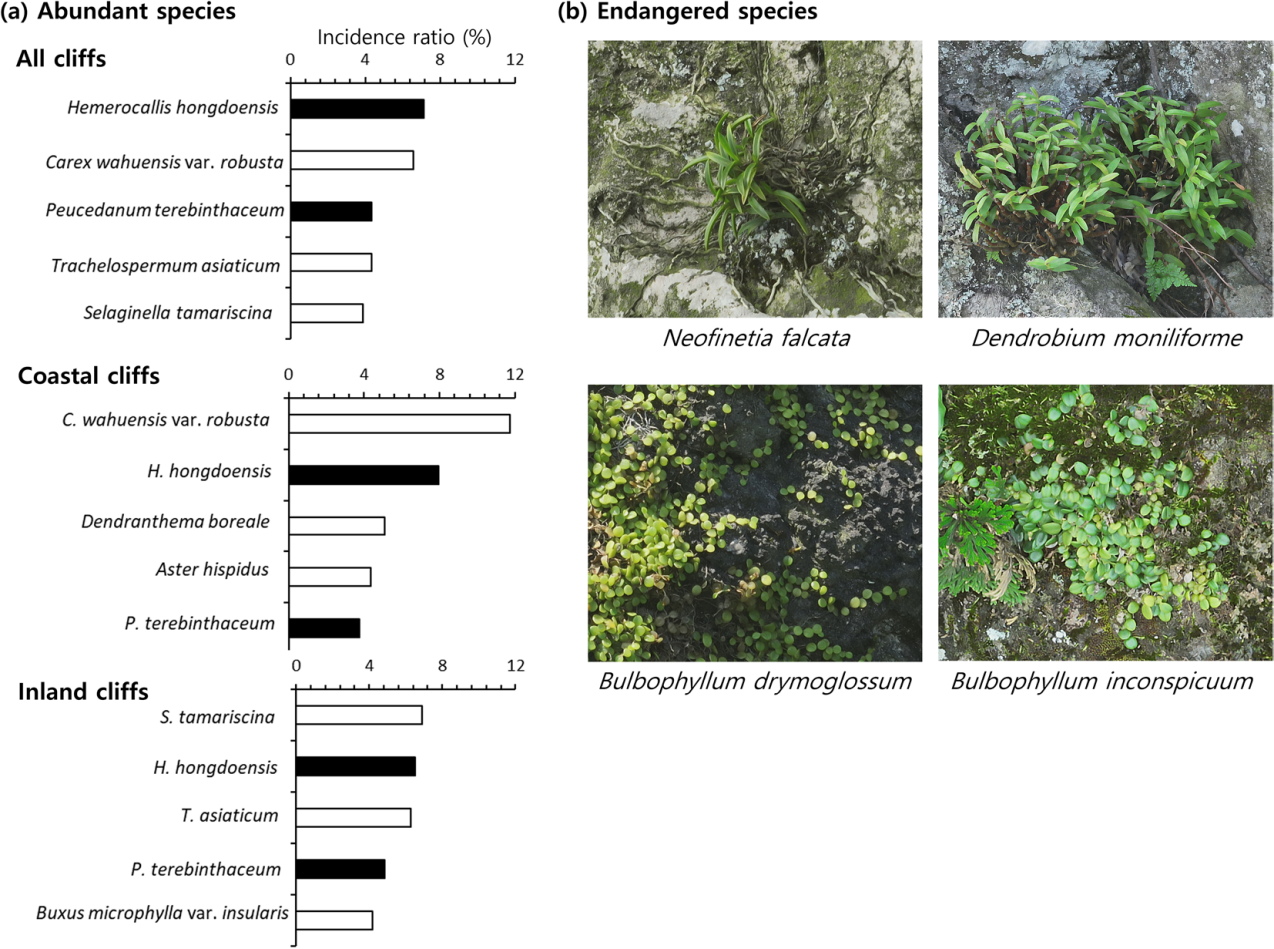
Incidence ratio of the abundant plant species (**a**) and UAV telephoto images of endangered plant species detected from the studied cliffs (**b**). Black bars in panel (**a**) infer the species abundant at both cliff types. Incidence ratio indicates the percentage of counted individuals for each species within the total amount of the individuals (3841 for coastal cliffs, 4620 for inland cliffs).

A total of 203 plant species from 81 families were detected throughout all studied cliffs (Table A1). Asteraceae (24 species), Poaceae (16 species), Rosaceae (8 species), Asparagaceae (7 species), and Crassulaceae (6 species) were the most prevalent families, accounting for 30.0% of the detected plant species. *Hemerocallis hongdoensis* (7.2%) occupied the highest incidence ratio, followed by *Carex wahuensis* var. *robusta* (6.6%) and *Peucedanum terebinthaceum* (4.3%) (Fig. 1a). If based on the cliff type, coastal and inland cliffs contained 154 and 166 species, respectively. *C*. *wahuensis* var. *robusta* (11.7%) and *Selaginella tamariscina* (6.9%) showed the highest incidence ratio in coastal and inland cliff types, respectively (Fig. 1a). There were four endangered, four Korea endemic, and 17 alien species in the studied cliffs (Table A1). Of the four endangered species (Fig. 1b), *Neofinetia falcata* was found exclusively in one of the coastal cliffs, while *Dendrobium moniliforme*, *Bulbophyllum drymoglossum*, and *Bulbophyllum inconspicuum* were observed exclusively in the inland cliffs. All these four endangered species were protected as CITES appendix II. Full list of the detected species is provided in Table A1.

**Figure 2:**
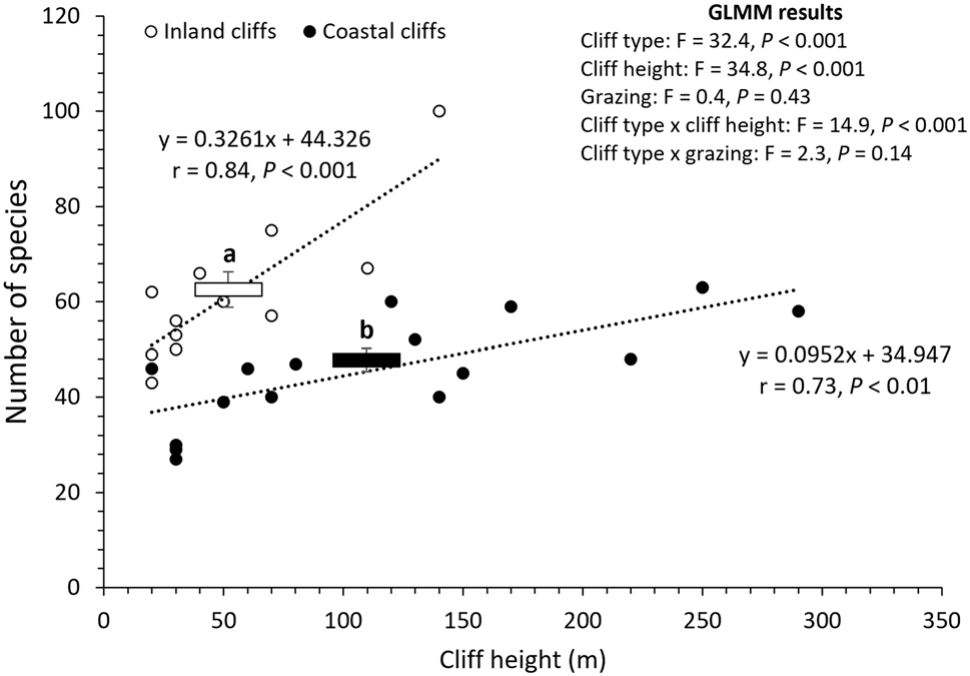
Comparison of the number of detected plant species between coastal and inland cliffs. Box graph with vertical error bars indicates average value and standard error (n = 17 for coastal cliffs and 13 for inland cliffs). Dots and dashed lines show the relationship between the number of plant species and cliff height. Letters above the box graph inform statistically significant difference based on Tukey’s HSD test (*P* < 0.05).

Generalized linear mixed model (GLMM) demonstrated that cliff type (*P* < 0.001), cliff height (*P* < 0.001), and their interaction (*P* < 0.001) had a significant effect on the number of detected plant species (Fig. 2). Grazing had no significant effect on the number of detected plant species (*P* = 0.43). The inland cliffs generally involved more plant species than the coastal cliffs, although the number of detected plant species increased with cliff height (r = 0.73–0.84). The gradient of linear regression between the number of detected plant species and cliff height was steeper in the inland cliffs than in the coastal cliffs (Fig. 2). Meanwhile, cliff type (*P* < 0.001) and cliff height (*P* < 0.001) had a significant effect on Shannon diversity index, while grazing and their interactions had no significant effect (*P* = 0.07–0.33). Shannon diversity index (mean ± standard error) was generally higher in the inland cliffs (4.10 ± 0.06) than in the coastal cliffs (3.79 ± 0.06).

**Figure 3:**
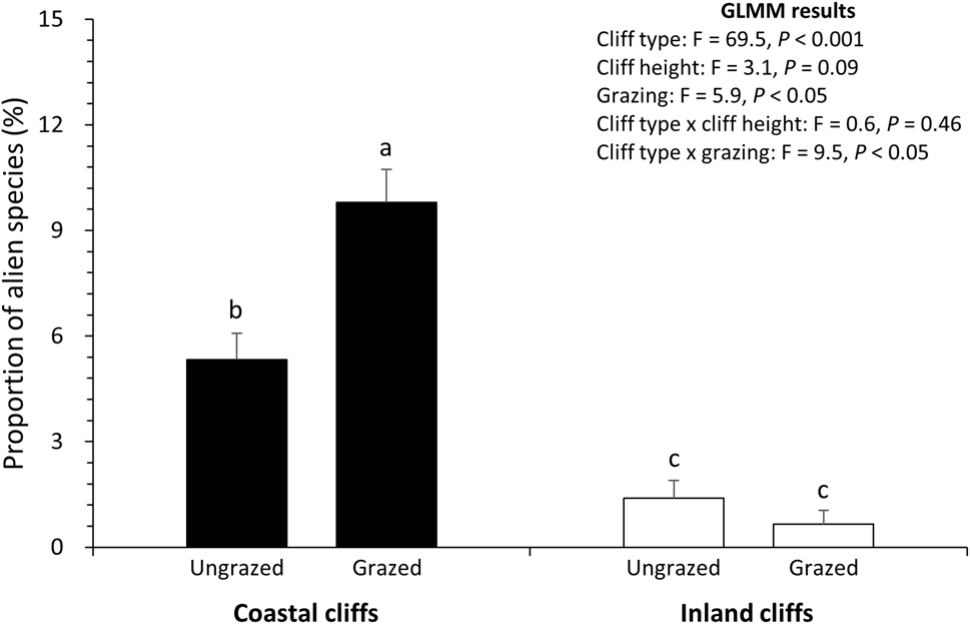
Proportion of alien species of coastal and inland cliffs with or without grazing. Error bars indicate standard error. Letters above the error bars inform statistically significant difference based on Tukey’s HSD test (*P* < 0.05) (n = 8 for ungrazed coastal cliffs, 9 for grazed coastal cliffs, 8 for ungrazed inland cliffs, and 5 for grazed inland cliffs).

Of the 17 alien plant species, 16 and 7 were found in the coastal and inland cliffs, respectively (Table A1). The GLMM also exhibited that cliff type (*P* < 0.001), grazing (*P* < 0.05), and their interaction (*P* < 0.05) had a significant effect on the proportion of alien species (Fig. 3). Cliff height had no significant effect on the proportion of alien species (*P* = 0.09). The coastal cliff (5.3–9.8%) generally had the higher proportions of alien species than the inland cliffs (0.6–1.4%). Grazing elevated the proportion of alien species only in the coastal cliffs rather than the inland cliffs (Fig. 3), reflecting that the coastal cliffs were potentially more vulnerable to grazing interventions than the inland cliffs.

### Lifeform composition

The coastal cliffs featured the higher proportions of herbaceous (annual and perennial) species but lower proportions of fern and woody (deciduous and evergreen) species than the inland cliffs (Table 2). The GLMM demonstrated that cliff type (*P* < 0.001), grazing (*P* < 0.01) and its interaction (*P* < 0.001) had a significant effect on the proportion of annual herb species (Table 2). The post-hoc test also showed that grazing increased the proportion of annual herb species only in the coastal cliffs, while such difference from grazing was not detected in the inland cliffs. Conversely, only cliff type (*P* < 0.05) had a significant effect on the proportions of other lifeforms while the effect of grazing and cliff height had no consistent pattern.

**Table 1:** Proportion of herbaceous (annual or perennial), fern, and woody (deciduous or evergreen) species in the studied coastal and inland cliffs.

		Coastal cliffs		Inland cliffs	
		Ungrazed	Grazed	Ungrazed	Grazed
Annual (%)		12.8 (1.6) b^1^	17.2 (0.5) a	6.7 (0.5) b	6.1 (1.3) b
GLMM^2^	F-value	T: 81.1, H: 0.4, G: 7.7, T × H: 0.9, T × G: 10.2			
	* P* value	T: < 0.001, H: 0.53, G: < 0.01, T × H: 0.35, T × G: < 0.001			
Perennial (%)		49.6 (1.4) ab	53.1 (2.4) a	43.5 (1.6) bc	39.8 (3.1) c
GLMM	F-value	T: 19.1, H: 3.3, G: 0.2, T × H: < 0.1, T × G: 1.2			
	* P* value	T: < 0.001, H: 0.08, G: 0.69, T × H: 0.94, T × G: 0.28			
Fern (%)		4.1 (1.2) b	3.9 (0.9) b	8.6 (0.7) a	8.0 (1.8) a
GLMM	F-value	T: 16.3, H: 1.0, G: < 0.1, T × H: 0.8, T × G: 0.4			
	* P* value	T: < 0.001, H: 0.33, G: 0.99, T × H: 0.39, T × G: 0.53			
Deciduous (%)		13.9 (1.7) ab	10.2 (1.9) b	20.3 (2.1) a	22.0 (3.7) a
GLMM	F-value	T: 15.8, H: 0.1, G: 0.6, T × H: < 0.1, T × G: 2.0			
	* P* value	T: < 0.001, H: 0.83, G: 0.46, T × H: 0.99; T × G: 0.17			
Evergreen (%)		19.6 (1.1) ab	15.6 (2.0) b	20.7 (1.4) ab	24.0 (3.0) a
GLMM	F-value	T: 5.6, H: 2.0, G: < 0.1, T × H: < 0.1, T × G: 2.4			
	* P* value	T: < 0.05, H: 0.17, G: 0.98, T × H: 0.91, T × G: 0.14			

^1^Lowercase letters indicate statistically significant difference based on Tukey’s HSD test (*P* < 0.05) (n = 8 for ungrazed coastal cliffs, 9 for grazed coastal cliffs, 8 for ungrazed inland cliffs, and 5 for grazed inland cliffs).

^2^Results of generalized linear mixed model (GLMM): T: cliff type, H: cliff height, G: grazing.

**Table 2:** Information of the studied cliff areas.

Cliff type	Coastal cliffs	Inland cliffs
Number of cliffs		
Grazed	9	5
Ungrazed	8	8
Altitude (m)		
Cliff top^a^	112 (20)	378 (29)
Cliff foot	0 (0)	325 (34)
Cliff height (m)^b^		
Average	112 (20)	53 (10)
Minimum	20	20
Maximum	290	140
Topography	Cliff foot is directly exposed to the ocean surface	Cliff foot is surrounded by the vegetated land

^a^Values in the parenthesis indicate standard errors.

^b^Distance between cliff foot and cliff top.

**Figure 4:**
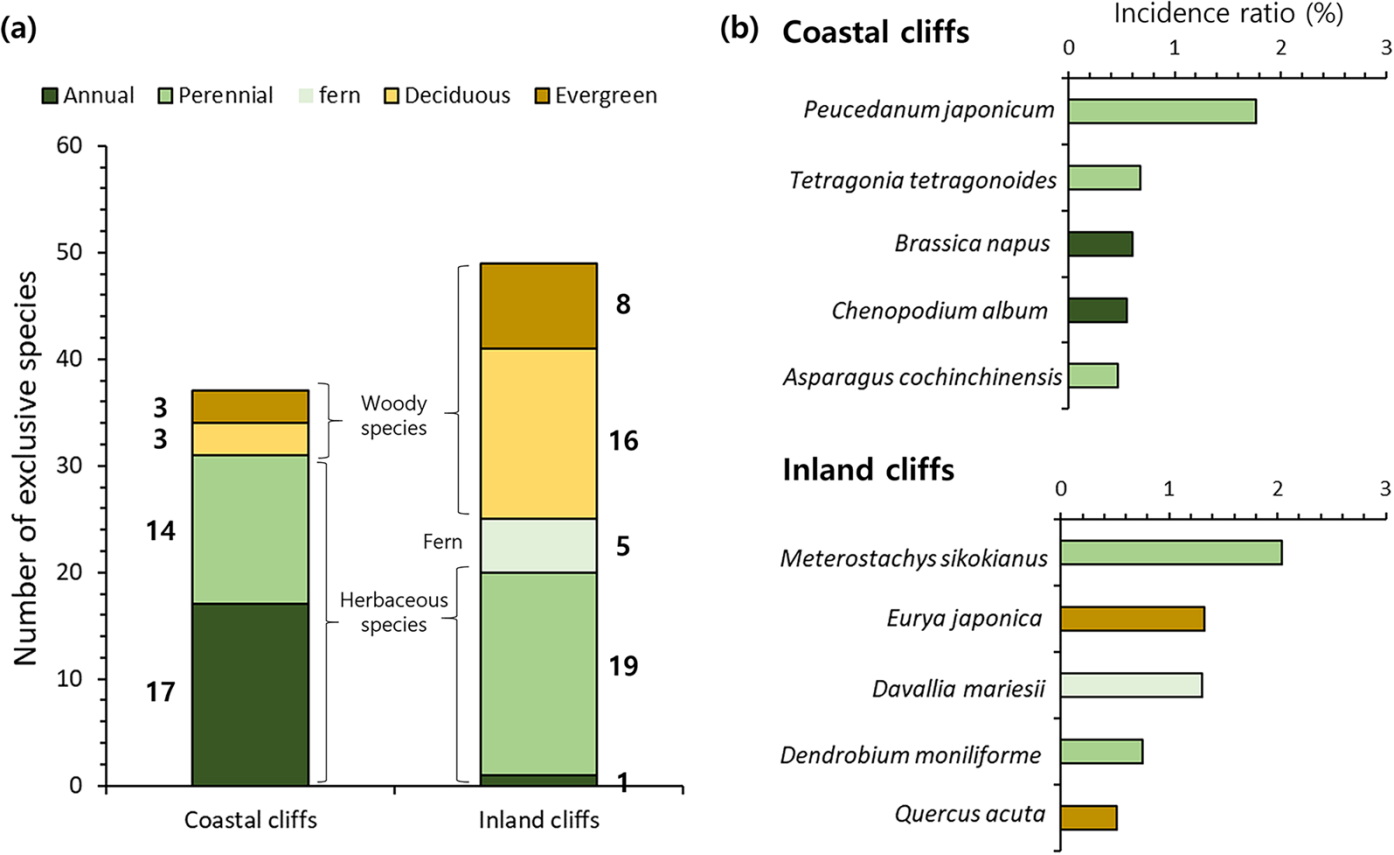
Number of plant species exclusively detected in coastal or inland cliffs (**a**) and incidence ratio of the major species exclusively detected in either cliff type (**b**). Incidence ratio indicates the percentage of counted individuals for each species within the total amount of the individuals (3841 for coastal cliffs, 4620 for inland cliffs).

Herbaceous (annual: 45.9%, perennial: 37.8%) species accounted for the largest proportion within the 37 species exclusively detected in the coastal cliffs, and no fern species was exclusive to the coastal cliff (Fig. 4a). Inversely, deciduous woody species (32.7%) recorded the largest proportion within the 49 species exclusively detected in the inland cliffs, and only 1 annual herb species was exclusive to the inland cliffs (Fig. 4a). All 5 major exclusive species (based on incidence ratio) were herbaceous in the coastal cliffs, whereas 2, 2, and 1 of them were perennial herb, evergreen woody, and fern species in the inland cliffs, respectively (Fig. 4b).

### Species composition and beta diversity

**Figure 5:**
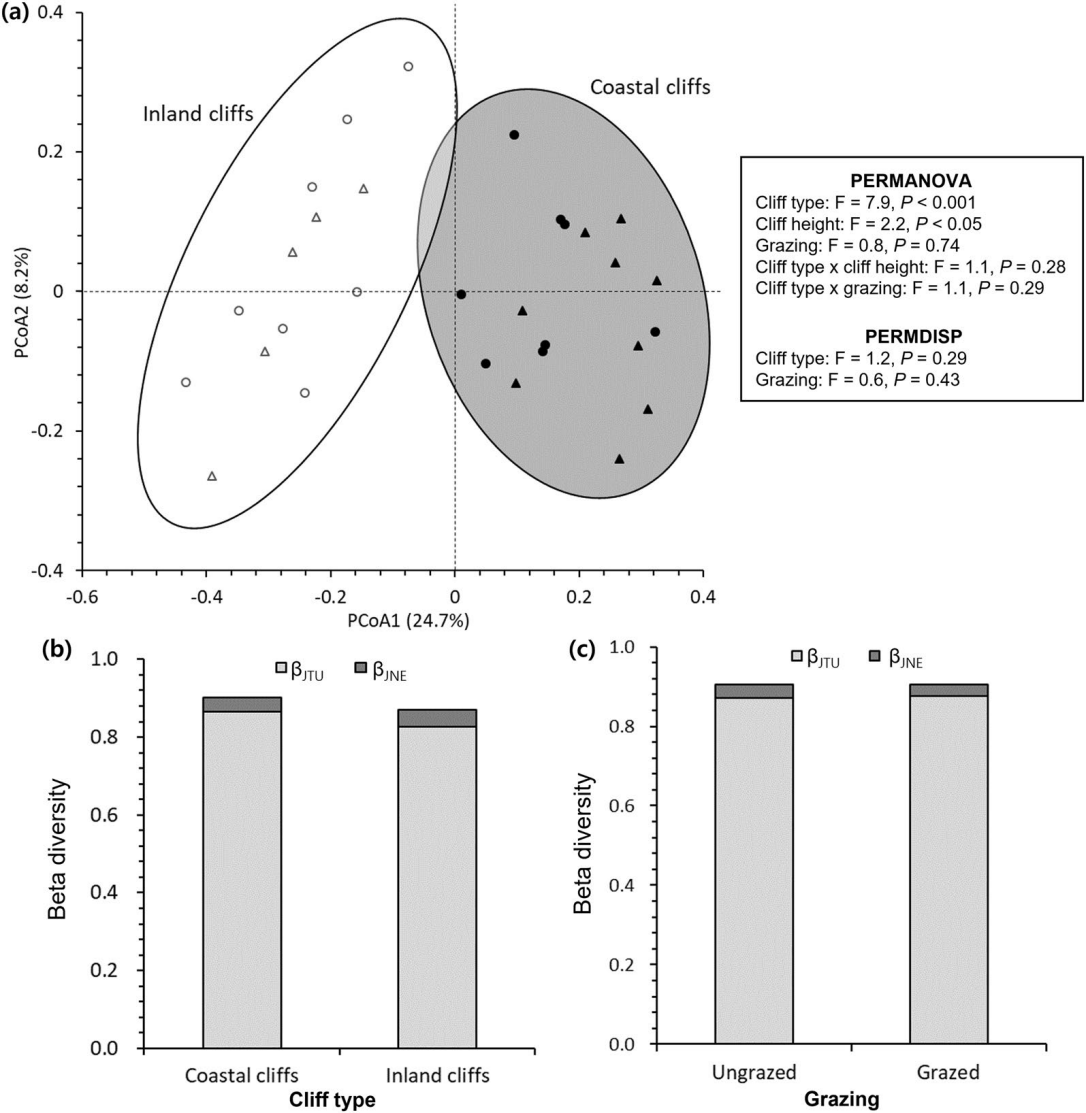
Principal coordinates analysis (PCoA), permutational multivariate analysis of variance (PERMANOVA), and permutational analysis of multivariate dispersion (PERMDISP) on beta diversity with Jaccard dissimilarity index (**a**) and proportion of turnover (β_JTU_) and nestedness-resultant (β_JNE_) components for the beta diversity according to cliff type (**b**) and grazing (**c**). Color and shape of dots in panel (**a**) represent cliff type (coastal cliff: black, inland cliff: white) and grazing (grazed: triangle, ungrazed: circle), respectively. PERMANOVA and PERMDISP are based on 9999 permutations.

First, second, and third axes of principal coordinates analysis (PCoA) explained 24.7, 8.2, and 7.3% of multivariate dispersions of the species composition, respectively (Fig. 5a). While the first axis clearly separated the inland cliffs from the coastal cliffs, the second axis slightly reflected the variations between grazed and ungrazed cliffs (Fig. 5a). The third axis was correlated to cliff height (r = 0.67, *P* < 0.01). Permutational multivariate analysis of variance (PERMANOVA) showed that cliff type (*P* < 0.001) and cliff height (*P* < 0.05) had a significant effect on the multivariate group centroids of species composition (Fig. 5a). However, permutational analysis of multivariate dispersion (PERMDISP) reported no significant effect of cliff type (*P* = 0.29) and grazing (*P* = 0.43) on the multivariate dispersions of species composition (Fig. 5a). Consistently, the beta diversity was similar across the cliff types and grazing conditions, which was mainly attributed to turnover component (β_JTU_: 0.826–0.876) instead of nestedness resultant component (β_JNE_: 0.028–0.043) (Fig. 5b, c).

## Discussion

### Effects of cliff type

Regarding the first hypothesis, our results show that the inland cliffs contained more vascular plant species than the coastal cliffs (Fig. 2). Flora of the two cliff types also differed in lifeform composition, among which higher proportions of fern and woody species were found in the inland cliffs than in the coastal cliffs (Table 1). This might occur because inland cliffs potentially experience plant migration from the surrounding non-cliff forests and grasslands^5^, whereas the ocean could physically prevent such species input to coastal cliffs. Direct salt deposition by the ocean spray could also decrease the fitness of any plants without enough salt-tolerance^1,30^, by which only limited number of species could survive at microhabitats within the coastal cliffs compared to those within the inland cliffs.

**Figure 6:**
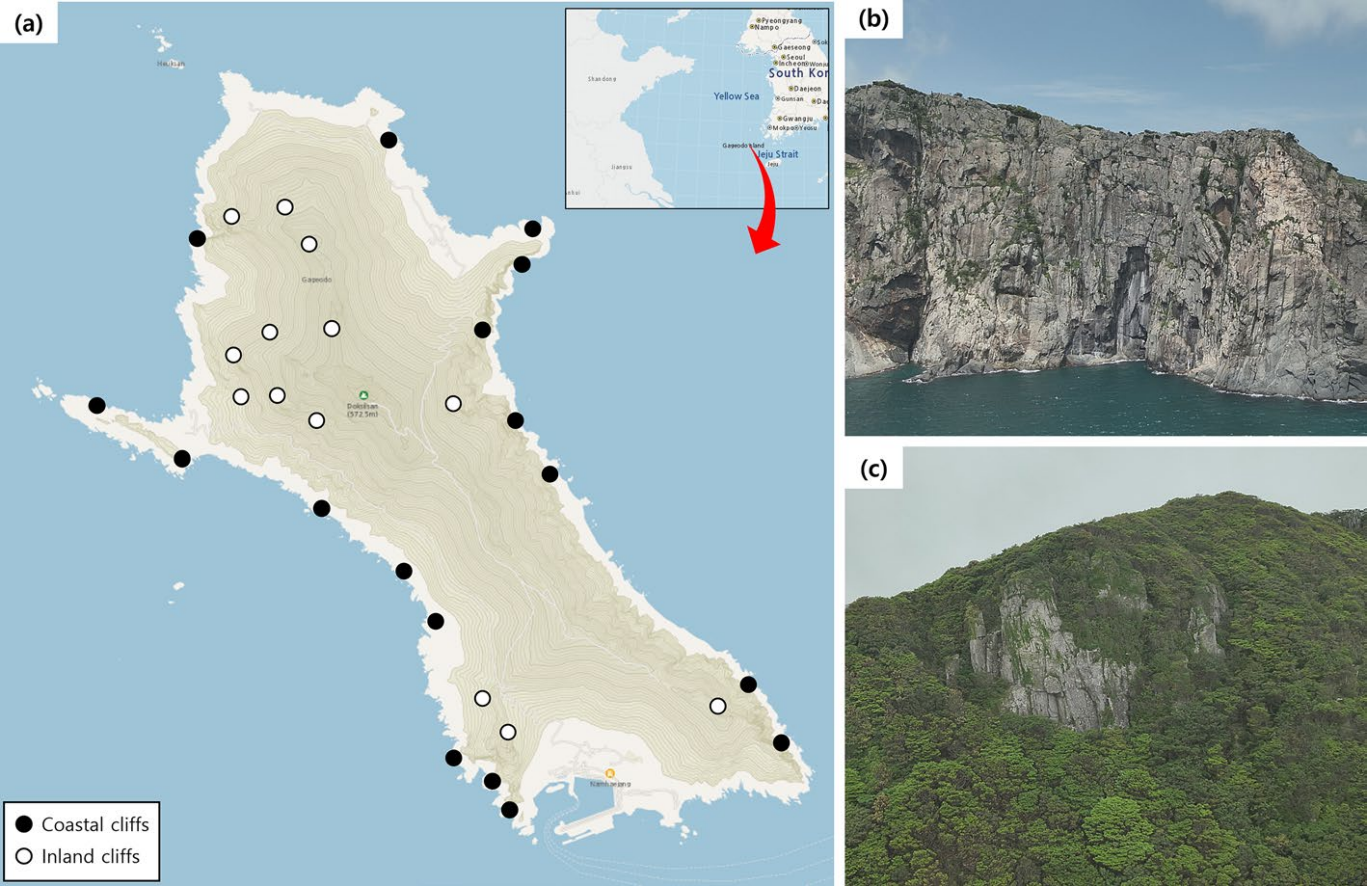
Location of the studied cliff areas (**a**) and examples of coastal (**b**) and inland cliffs (**c**). The map used in panel (**a**) is taken from the open-source database of National Geographic Information Institute of South Korea (Source: https://map.ngii.go.kr/ms/map/NlipMap.do, accessed on 8 December 2023).

The two cliff types were further distinguishable in terms of plant species composition, reflected by the different multivariate group centroids (Fig. 5a). It was found that only the flora of the coastal cliffs was characterized by the abundance of halophytic species such as *C. wahuensis* var *robusta*, *Aster hispidus*, *Peucedanum japonicum*, and *Tetragonia tetragonoides* (Figs. 3a, 6b)^33^. This pattern confirms the potential differentiation of plant communities between the coastal and inland cliffs according to the salt-tolerance^1^. Conversely, the inland cliffs featured the abundance of an epiphytic fern (*S. tamariscina*, Fig. 1a) and 5 fern species undetected in the coastal cliffs (Fig. 4a). Considering that epiphytes on the cliffs strongly rely on substrate conditions^16^, such difference indicates that saline microhabitats within the coastal cliffs might lower the fitness of epiphytic ferns.

Another interesting finding is the effects of cliff type being dependent on cliff height. Particularly, the difference in the number of plant species between the coastal and inland cliffs was magnified with increasing cliff height (Fig. 2), although elevated cliff height provides larger surface for the survival of diverse plant species regardless of the cliff type^8,30^. In fact, coastal cliffs are known by their susceptibility to mass waste by the direct exposure to tidal impacts from the ocean^34^ and to soil erosion at the cliff top by rainfall and wind^1,35^. These extreme disturbances around coastal cliffs might reduce the availability of stable microhabitats to establish plant communities relative to inland cliffs^18,36^, and accordingly induce the slower increase in the number of plant species with the height of the coastal cliffs.

The multivariate dispersions of species composition, however, did not vary between the cliff types (Fig. 5a), inferring no significant differentiation in the taxonomic beta diversity^37^. This pattern is unexpected because the gradients of salt deposition are known to affect plant beta diversity along the coasts^38,39^. Such lack of the differences in the beta diversity possibly occurred because both the studied cliff types have a similar annual precipitation and parent material of Gageodo island^40^, which are the major factors controlling moisture availability and beta diversity of cliff plant communities^16,41^. The dominance of β_JTU_ in both the cliff types also assists this idea (Fig. 5b), considering that the turnover components of beta diversity are the function of spatial, environmental variations within each group of comparison, not only the difference in species composition^42^.

### Effects of grazing interventions

Anthropogenic introduction of herbivores has threatened island plant communities as it frequently leads to overgrazing, soil degradation, and disposal of invasive plant species^43–45^. In this context, cliffs in island ecosystems are considered as refuges for native plants to avoid such threats from the anthropogenic changes^30,46^. Regarding the second hypothesis, our results demonstrate that the grazed coastal cliffs involved the increased proportion of alien plant species than the ungrazed coastal cliffs (Fig. 3). Similar pattern was also observed by the elevated proportion of annual herb species under grazing of the coastal cliffs (Table 1). These patterns are in line with Thomson et al.^45^, who informed the invasion of non-native, fast-growing species and the suppression of native, slow-growing species by introduced herbivores in island plant communities. Our findings further suggest that cliff plant communities might not be immune to biological invasion and grazing if the introduced herbivores are able to access the cliff microhabitats. It supports previous reports on the alterations in cliff ledge flora after inclusion of cliff-dwelling ungulates^13^.

Interestingly, the effects of grazing were undetected in the inland cliffs (Fig. 3, Table 1), illustrating different vulnerabilities to the cliff-dwelling herbivores between the two cliff types. It might be related to the proximities to coast because repetitive natural disturbances around coastal areas promote the competitive replacement and invasibility of alien plant species^47,48^. This unstable environment might boost the spread of fast-growing, short-living, and non-native species only into the coastal cliffs under grazing interventions^45^. The relatively high proportion of woody species in the inland cliffs might also counteract the effects of grazing (Table 1), given that invasion into the woody plant communities requires the sufficient shade-tolerance^49^.

**Figure 7:**
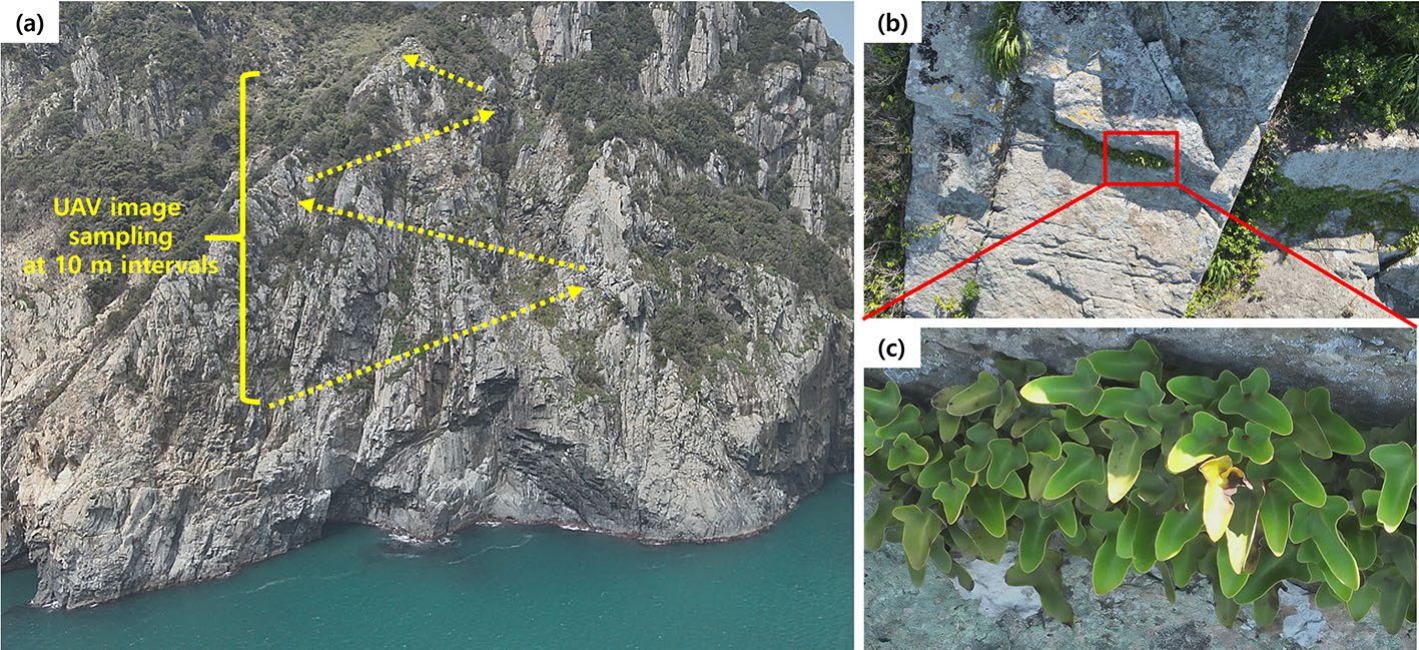
Examples of transects to collect UAV image data (**a**), UAV images taken by wide-angle lens (**b**) and by telephoto lens for detailed species identification (**c**; *Pyrrosia hastata*).

There was no distinct change in the species composition, beta diversity, and number of detected species under grazing in both the cliff types (Figs. 4, 7), unlike the proportions of annual herb and alien species. Our results are inconsistent with the previous reports on the negative impacts of introduced herbivores and alien species invasion on plant diversity across cliffs^11^ and other island ecosystems^45,50^. It reflects that the magnitude of such interventions across the studied cliffs might not be heavy enough to distort the overall taxonomic structure and beta diversity at the current stage. Otherwise, no effect on the species composition and beta diversity might occur as decrease in native plant diversity by biological invasions becomes more distinct at fine quadrate-scale investigations than landscape-scale approaches^32^. As the effects of grazing interventions could be altered by grazing intensity and elapsed time^13,44^, long-term, repetitive monitoring should be necessary to ensure whether the species composition and beta diversity will remain unchanged in the future.

### Implications

The overall results allow us to partly accept the null hypotheses regarding the effects of cliff types and grazing on cliff flora. The coastal and inland cliffs appeared to differ in the number of detected species, lifeform composition, the proportion of alien species, and overall species composition with no clear differentiation in the beta diversity. Similarly, the grazed and ungrazed coastal cliffs were distinguishable according to the proportions of alien and annual herb species. In terms of biodiversity conservation, these findings indicate that cliff plant communities along the coasts might be sensitive to biological invasion of alien plants^11^, which could be magnified by grazing of the introduced cliff-dwelling ungulates. In fact, the proportion of alien species was similar between the grazed coastal cliffs (9.8%) and non-cliff areas (8.9%)^51–54^, both of which were higher than those in the ungrazed coastal cliffs (5.3%) as well as the inland cliffs (0.6–1.4%). Considering that coastal cliffs of the neighboring, well-preserved island reserve featured the lower proportion of alien plant species (2.1%)^30^, the anthropogenically introduced herbivores should be excluded to conserve the native plant communities along the coastal cliffs. Given the high turnover components within the beta diversity across the studied cliffs (Fig. 5), we also suggest that individual cliffs potentially have unique plant communities requiring multi-site conservation strategies rather than protection of a single habitat with the highest species abundance^32^. As only one southeastern coastal cliff contained the habitat of the endangered species *N. falcata*, particular attentions should be paid to that cliff site to prevent excessive grazing and illegal poaching.

Beyond the null hypotheses, our results exhibit the usefulness of UAV to study plant communities on the inaccessible ecosystems. For example, our UAV telephotography found the epiphytic fern, *Pyrrosia hastata* from inaccessible cliff microhabitats (Fig. 7c in “Methods” section), though previous studies on traditionally accessible sites have informed that this species does not occur in the study area^51^. The endangered orchid species, *N. falcata* has also remained undetected after the last observation by Yun et al.^52^ until the present study (Fig. 1b). Furthermore, the obtained telephoto images showed the natural reproduction of *P.* *macrophyllus* populations on the unreachable coastal cliff faces (Fig. A2), supporting the idea that this subtropical conifer might be native to the study area^53^. These floristic findings affirm the necessity of exploring inaccessible microhabitats to fully shed light on regional biodiversity by overcoming the uncertainties from sampling bias to traditionally reachable plant communities, even though the studied inaccessible cliffs contained only 37.5% of the vascular plant species in the non-cliff sites^51^. Similar utilizations of the UAV telephotography will help improve the reliability of the original close-range UAV approaches^8,30^, especially when identifying small plant species under other challenging conditions.

Several future collaborations may reinforce the usefulness of UAV for ecological researches. Particularly, the attachment of mechanical samplers will enable to collect eDNA from inaccessible ecosystems for the detailed phylogenetic analyses on any species without noticeable morphological traits^27,28^. Developing vertical auto flight methodologies may be helpful to reduce time–cost and enhance long-term monitoring consistency compared to the current UAV approaches based on manual flight^23^. The use of multispectral UAV sensors will deepen our understanding of plant ecophysiology at the harsh, challenging microhabitats, and relieve the confounding effects of spatial sampling scale by comparing with satellite imageries and ground-level observations^9^. Although grazing intensity can be an important factor controlling the cliff vegetation^43^, our study on the inaccessible cliffs did not quantitatively account for the grazing intensity. Thus, future studies should consider the gradients of herbivore population sizes and the associated grazing intensities for experimental design. Wind exposure, aspect, and other microtopographic factors may also be other critical factors influencing the floristic patterns across cliffs^30^, which further UAV investigations should take into consideration.

## Conclusions

Cliffs are one of the least known ecosystems because of the inherent inaccessibility and harsh environmental conditions. Here, the UAV telephotography was adopted to overcome such challenges and describe its applicability to clarifying the influential factors for cliff plant communities. Results show that plant communities in the coastal cliffs featured the lower number of species, higher proportions of herbaceous and alien species, and the abundance of halophytic species than those in the inland cliffs. These differences between the cliff types are more on the overall species composition of plant communities (multivariate group centroids) rather than the beta diversity (multivariate group dispersion). In addition, grazing of the introduced goats elevated the proportions of annual herbs and alien species especially in the coastal cliffs. This indicates that coastal cliffs might be not totally immune to the anthropogenic interventions by grazing, requiring the appropriate exclusion treatments of the introduced cliff-dwelling herbivores. We expect that similar uses of the UAV telephotography will enhance our understanding of cliff plant communities as well as other unstudied ecosystems because of the extreme inaccessibility.

## Methods

### Study area

Studied cliff areas are located in Gageodo, the island at the southwesten end of South Korea (34°04′N, 125°06′E) (Fig. 6a). Distance from the main Korean peninsula is 127 km, and the average annual temperature and precipitation are 13.8 °C and 1120 mm^51^. Size of the island is approximately 9 km^2^, with the maximum altitude of 639 m^54^. The coasts of Gageodo feature the steep cliffs directly exposed to the ocean surface (Fig. 6b), and 95.8% of the lands comprise mountainous forests with rocky cliffs and ledges (Fig. 6c)^55^. The parent materials are igneous rocks, mainly consisting of andesite with a minor proportion of rhyolite^40^.

Natural vegetation of the inner mountains in Gageodo is characterized as broadleaved evergreen forests under the warm-temperate climate, which is dominated by *Castanopsis sieboldii* and *Machilus thunbergii*^56^. The latest studies on the accessible, non-cliff areas reported 541 vascular plant species^51^, of which 11, 6, and 48 were Korea endemic, endangered, and alien plant species, respectively^52–54^. This island is the northernmost distributional limit of the evergreen shrub, *Viburnum japonicum*^52^, and debatably considered as the only natural Korean habitat of the subtropical conifer, *Podocarpus macrophyllus*^53^. However, the vegetation of Gageodo has been impacted by anthropogenic interventions, such as overgrazed goats and cattle, recreational fishing, and long-term breakwater construction^55^. Due to the absence of native predators, overgrazed goats were accidentally naturalized throughout the entire island, and become invasive even to cliff and crag landscapes (Fig. A1).

### Studied cliffs and UAV investigations

A UAV (Mavic 3 Enterprise, DJI, China) containing wide-angle (48 megapixel sensor with f/2.8 lens) and telephoto (12 megapixel sensor with f/4.4 lens; up to 7× optical and 56× hybrid zooms) cameras was utilized in the present study. This UAV weighs approximately 920 g and can fly up to 45 min. There are infrared and omni-directional sensing systems to detect surrounding obstacles for flight safety. It is also equipped with a real time kinematic (RTK) module, ensuring the positioning and hovering accuracies of 1 cm ± 1 ppm and ± 0.1 m for horizontal direction and 1.5 cm ± 0.1 ppm and ± 0.1 m for vertical direction, respectively.

Our UAV investigations targeted on a total of 30 cliff sites with different cliff types and grazing conditions (Table 2). Of them, 17 were categorized as the coastal cliffs that was directly exposed to the ocean surface (Fig. 6b), while the others were classified as the inland cliffs that was sheltered by vegetated mountainous lands (Fig. 6c)^1^. The studied cliffs were subdivided in accordance with the occurrence and feeding trait of cliff-dwelling goat populations (Fig. A1) to address the effect of grazing by the introduced herbivores (Table 2). Here, feeding traits and damages by goats in the UAV image data (e.g., Fig. A1a, b) were considered as the occurrence of the cliff-dwelling goat populations, to minimize potential bias due to the temporal absence of the herbivores during the UAV investigations. Height of the studied cliffs were 20–290 m for the coastal cliffs and 20–140 m for the inland cliffs, respectively (Table 2).

UAV image was sampled three times (April, June, and August 2023) for each cliff to collect clear morphological data of the detected plant species. The UAV image was taken through the multiple diagonal transects on cliff face area (Fig. 7a), as described by^30^. A wide-angle lens photograph was taken for each sampling point at 3–6 m away from the cliff surface (safe distance to protect UAV)^8^ (Fig. 7b), and additional photographs were taken using the telephoto lens for any plants requiring the detailed species identification (Fig. 7c). Then, the UAV flew 10 m diagonally upward following the transects and hovered until stabilization for image samplings at the next sampling point. Such procedure was continued unless the UAV reached the cliff top area.

### Species identification and categorization

Species identification for UAV images was based on Zhou et al.^8^ and Kim et al.^30^. Three regional botanical experts independently identified the plant species within each wide-angle lens image by using the associated telephoto lens images. The experts primarily identified the detected plants into the species level; however, if there was no clear morphological trait data during all three sampling times, the taxa were classified up to the genus level only (1 species in the present study, Table A1). When the experts recorded different names for the same species, the morphological traits from the telephoto lens images were rechecked according to the botanical specimen studies on Gageodo^52–54^ and the standard illustration of Korean plants^57,58^. International Plant Name Index (IPNI) and World Checklist of Selected Plant Families (WCSP) were used for the taxonomy and nomenclature of the detected plant species. In addition, lifeform of the detected plant species was divided into herbaceous (annual or perennial), fern, and woody (deciduous or evergreen) species as described by the latest study on Gageodo^51^ for ease of direct comparisons. The occurrence of endangered species was checked using the list of the Ministry of Environment of South Korea, and Alien (archaeophyte and naturalized plants of Kang et al.^59^) and Korea endemic^60^ species were informed as well (Table A1).

### Data analyses

Incidence ratio was calculated to quantify the relative abundance for each plant species as follows^8,30^:

$$Incidence\;ratio \left( \% \right) = \frac{{I_{x} }}{{I_{total} }} \times 100$$

where I_x_ and I_total_ represent the number of incidences of plant individuals for a given species and total number of plant incidences throughout all analyzed UAV images (coastal cliff: 3841, inland cliff: 4620). In the present study, such incidence of plant individuals was counted once for each species per each wide-angle lens photograph (i.e., sampling point) because UAV image data provided no belowground information to confirm the actual number of individuals ^8^. Shannon diversity index was also estimated using the vegan^61^ package in R 4.2.1 (R Core Team, 2022) to enable direct comparisons of the obtained alpha diversity with traditional studies regarding plant diversity.

Normality and homoscedasticity were tested by Shapiro–Wilk and Leven’s tests for the number of detected species and the proportions of alien species and each lifeform, given the unbalanced experimental design (n = 8 for ungrazed coastal cliffs, 9 for grazed coastal cliffs, 8 for ungrazed inland cliffs, and 5 for grazed inland cliffs) (α = 0.05). Leven’s test confirmed the homogeneity of variance for all variables (*P* > 0.05); however, the proportion data were square root-transformed for normalization considering the result of Shapiro–Wilk test (*P* < 0.05). Then, generalized linear mixed model (GLMM) and Tukey’s HSD test were conducted to assess the effects of the two categorical variables (cliff type and grazing), one continuous variable (cliff height), and their interactions on the number of detected species and the proportions of alien species and each lifeform (α = 0.05). Pearson correlation and linear regression were also used to describe any pairwise relationships (α = 0.05). These analyses were performed using the Agricolae^62^ package in R 4.2.1 (R Core Team, 2022).

Taxonomic beta diversity with Jaccard dissimilarity index was estimated using the binomial occurrence data (absence: 0, presence: 1) of each species. Principal coordinates analysis (PCoA) with Jaccard dissimilarity was carried out to display multivariate patterns in the species composition and beta diversity. Permutational multivariate analysis of variance (PERMANOVA) and permutational analysis of multivariate dispersion (PERMDISP) with 9999 permutations were also conducted to evaluate the effects of cliff type, cliff height, and grazing on multivariate group centroids and dispersions (α = 0.05). Moreover, turnover (β_JTU_) and nestedness-resultant (β_JNE_) components within the beta diversity were separated to reveal the ratio between the dissimilarities associated with species replacements and losses. These analyses on the species composition and beta diversity were performed using vegan^61^, ecodist^63^, pairwiseAdonis^64^, and betapart^65^ packages in R 4.2.1 (R Core Team, 2022).

### Supplementary Information

Supplementary Information.

## Data Availability

Raw data used for statistical analyses are available from the first and corresponding authors. Full list of detected plant species is available at Table A1 in the attached supplementary materials file.
