# Supplementary material for: UAV telephotography elucidates floristic variability and beta diversity of island cliffs under grazing interventions
Source: Sci Rep. 2024 Jul 5;14:15465. doi: 10.1038/s41598-024-66446-7 (PMC11224387; doi:10.1038/s41598-024-66446-7)
Supplement: Supplementary file 1 — Supplementary Information. [file 41598_2024_66446_MOESM1_ESM.docx]

Supplementary materials

**UAV telephotography elucidates floristic variability and beta diversity of island cliffs under grazing interventions**

Seongjun Kim*, Chang Woo Lee*, Hwan-Joon Park, Jung Eun Hwang, Hyeong Bin Park, Young-Jun Yoon, Yeong-Joong Kim

Research Center for Endangered Species, National Institute of Ecology, Yeongyang 36531, Gyeongbuk Province, Republic of Korea

***Corresponding authors**

Seongjun Kim

Tel.: +82-54-680-7287

Fax: +82-54-680-7299

Email: [dao1229@nie.re.kr](mailto:dao1229@nie.re.kr)

Chang Woo Lee

Tel.: +82-54-680-7282

Fax: +82-54-680-7299

Email: jacky903@nie.re.kr


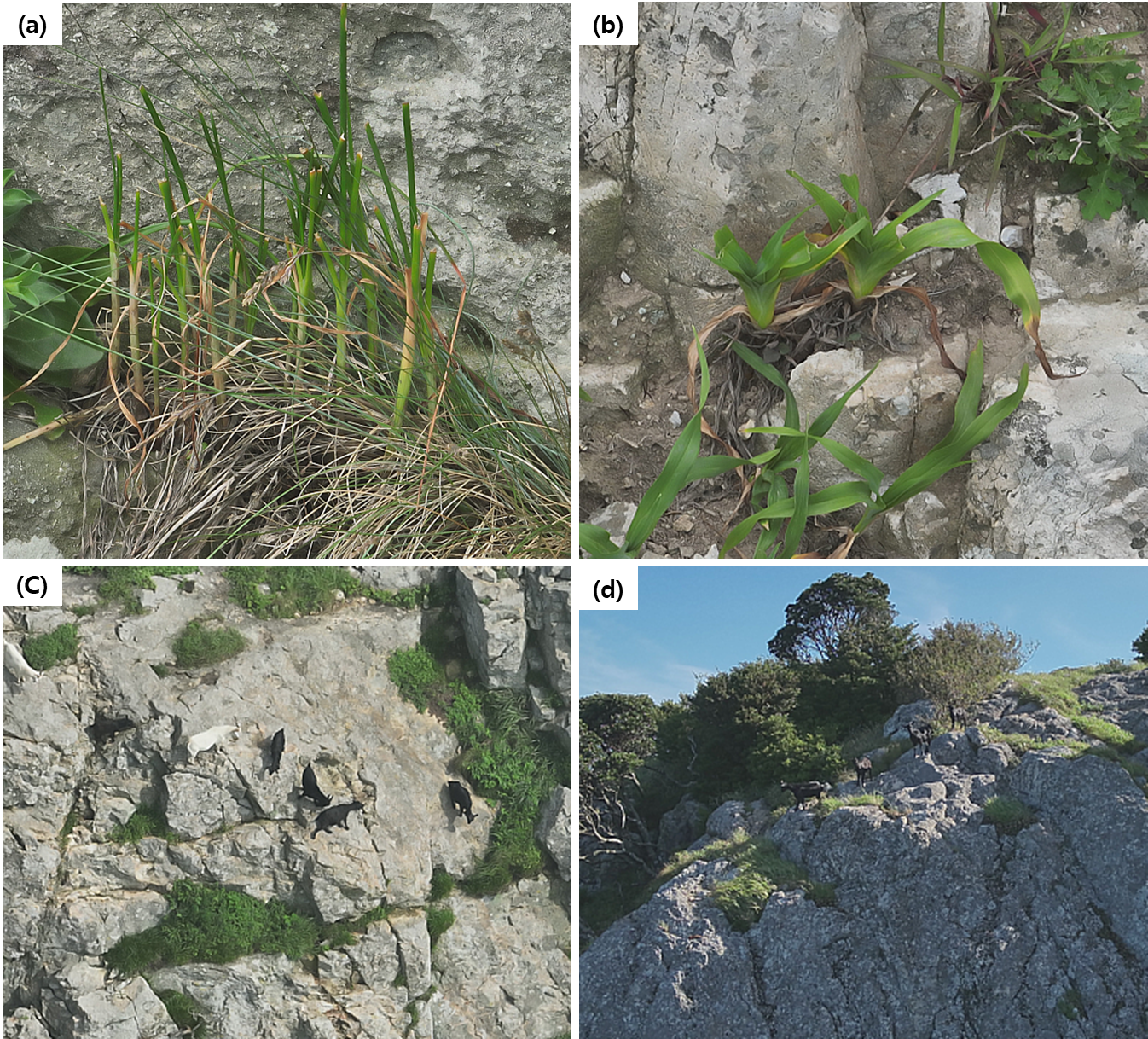


Fig A1. UAV images regarding the cliff vegetation disturbed by grazing (a, b) and the naturalized goat populations on the studied cliff areas (c, d).


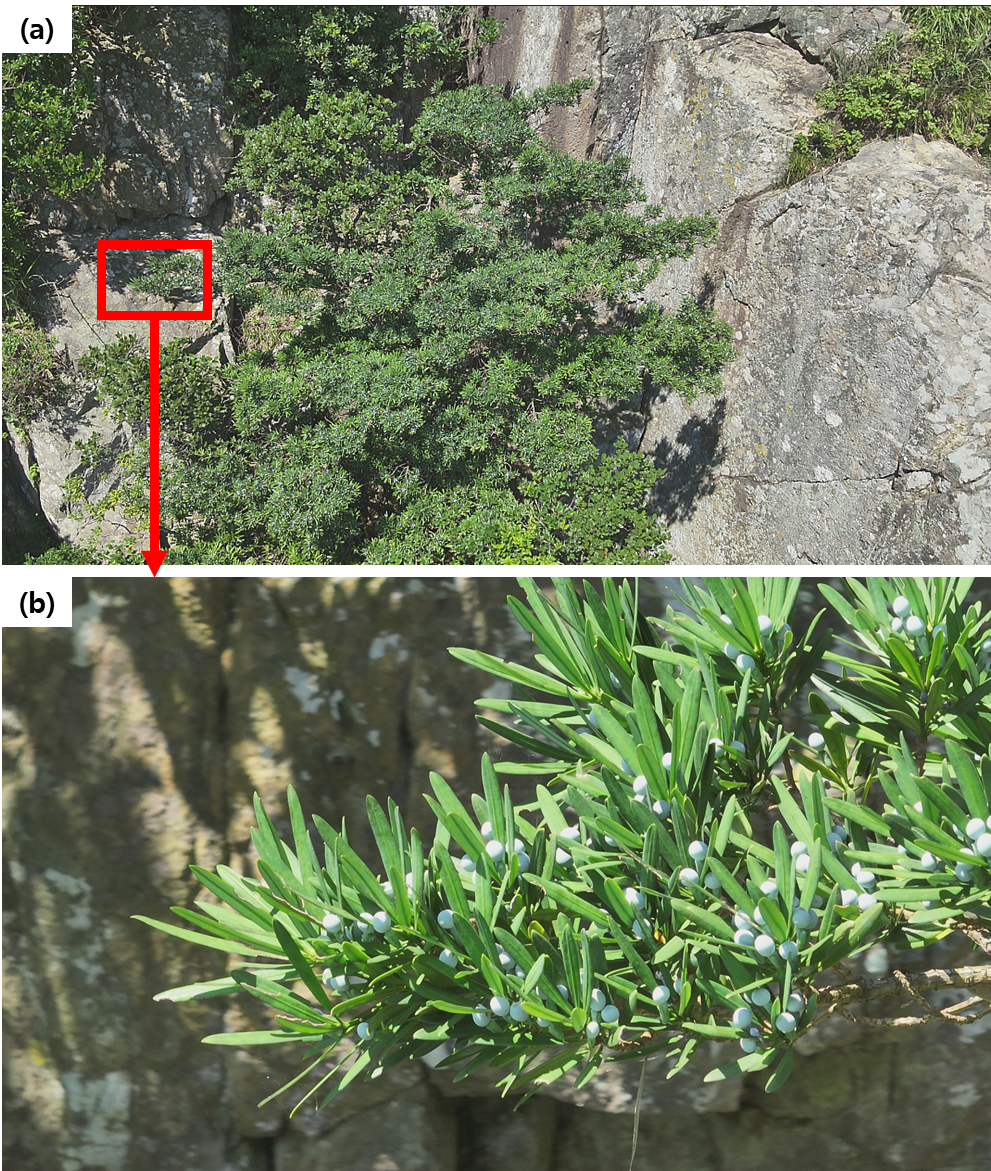


Fig A2. UAV images of the mature *Podocarpus macrophyllus* growing on the unreachable coastal cliff face (a: taken by wide-angle lens; b: taken by telephoto-lens)

Table A1. List of the detected plant species from the studied cliff areas.

| **Family** | **Scientific name** | **Incidence^1^** | | **Note^2^** |
| --- | --- | --- | --- | --- |
|  |  | **Coastal**  **cliff** | **Inland**  **cliff** |  |
| **Pteridophyta** | | | | |
| **Polypodiales** | | | | |
| Athyriaceae | *Athyrium yokoscense* (Franch. & Sav.) H. Christ | X | O |  |
| Davalliaceae | *Davallia mariesii* T. Moore ex Baker | X | O |  |
| Dennstaedtiaceae | *Pteridium aquilinum*var. *latiusculum* (Desv.) Underw. ex A. Heller | O | O |  |
| Dryopteridaceae | *Cyrtomium falcatum* (L. f.) C. Presl | O | O |  |
| Dryopteridaceae | *Dryopteris erythrosora* (D. C. Eaton) Kuntze | X | O |  |
| Dryopteridaceae | *Dryopteris saxifraga* H. Itô | O | O |  |
| Polypodiaceae | *Lemmaphyllum microphyllum* C. Presl | O | O |  |
| Polypodiaceae | *Lepisorus onoei* (Franch. & Sav.) Ching | O | O |  |
| Polypodiaceae | *Lepisorus thunbergianus* (Kaulf.) Ching | O | O |  |
| Polypodiaceae | *Pyrrosia hastata* (Thunb.) Ching | X | O |  |
| Polypodiaceae | *Pyrrosia lingua* (Thunb.) Farw. | X | O |  |
| **Selaginellales** | | | | |
| Selaginellaceae | *Selaginella tamariscina* (P. Beauv.) Spring | O | O |  |
| **Pinophyta** | | | | |
| **Cupressales** | | | | |
| Cupressaceae | *Juniperus procumbens* (Siebold ex Endl.) Miq. | O | O |  |
| **Pinales** | | | | |
| Pinaceae | *Pinus thunbergii* Siebold & Zucc. | O | O |  |
| **Podocarpales** | | | | |
| Podocarpaceae | *Podocarpus macrophyllus* (Thunb.) Sweet | O | X |  |
| **Magnoliophyta** | | | | |
| **Apiales** | | | | |
| Apiaceae | *Anthriscus sylvestris* (L.) Hoffm. | O | X |  |
| Apiaceae | *Hydrocotyle maritima* Honda | O | O |  |
| Apiaceae | *Ostericum sieboldii* (Miq.) Nakai | X | O |  |
| Apiaceae | *Peucedanum japonicum* Thunb. | O | X |  |
| Apiaceae | *Peucedanum terebinthaceum* (Fisch. ex Trevir.) Ledeb. | O | O |  |
| Araliaceae | *Aralia elata* (Miq.) Seem. | X | O |  |
| Araliaceae | *Hedera rhombea* (Miq.) Bean | O | O |  |
| Araliaceae | *Dendropanax trifidus* (Thunb.) Makino ex H. Hara | O | O |  |
| Pittosporaceae | *Pittosporum tobira* (Thunb.) W. T. Aiton | O | O |  |
| **Aquifoliales** | | | | |
| Aquifoliaceae | *Ilex integra* Thunb. | X | O |  |
| **Arales** | | | | |
| Araceae | *Arisaema ringens* (Thunb.) Schott | O | O |  |
| **Aristolochiales** | | | | |
| Aristolochiaceae | *Asarum sieboldii* Miq. | X | O |  |
| **Asparagales** | | | | |
| Amarylidaceae | *Allium sacculiferum* Maxim. | O | O |  |
| Asparagaceae | *Asparagus cochinchinensis* (Lour.) Merr. | O | X |  |
| Asparagaceae | *Barnardia japonica* (Thunb.) Schult. & Schult. f. | O | O |  |
| Asparagaceae | *Hosta yingeri* S. B. Jones | O | O | Ed |
| Asparagaceae | *Liriope platyphylla* F. T. Wang & T. Tang | O | O |  |
| Asparagaceae | *Ophiopogon jaburan* (Siebold) Lodd. | O | O |  |
| Asparagaceae | *Ophiopogon japonicus* (Thunb.) Ker Gawl. | O | O |  |
| Asparagaceae | *Polygonatum cryptanthum* H. Lév. & Vaniot | X | O |  |
| Asphodelaceae | *Hemerocallis hongdoensis* M. G. Chung & S. S. Kang | O | O | Ed |
| Colchicaceae | *Disporum smilacinum* A. Gray | X | O |  |
| Orchidaceae | *Amitostigma gracile* (Blume) Schltr. | X | O |  |
| Orchidaceae | *Bulbophyllum drymoglossum* Maxim. ex Ökubo | X | O | En |
| Orchidaceae | *Bulbophyllum inconspicuum* Maxim. | X | O | En |
| Orchidaceae | *Dendrobium moniliforme* (L.) Sw. | X | O | En |
| Orchidaceae | *Neofinetia falcata* (Thunb.) Hu | O | X | En |
| Smilacaceae | *Smilax china* L. | X | O |  |
| **Asterales** | | | | |
| Asteraceae | *Artemisia capillaris* Thunb. | O | O |  |
| Asteraceae | *Artemisia indica* Willd. | O | O |  |
| Asteraceae | *Aster ageratoides* Turcz. | O | O |  |
| Asteraceae | *Aster hispidus* Thunb. | O | O |  |
| Asteraceae | *Aster scaber* Thunb. | O | O |  |
| Asteraceae | *Aster spathulifolius* Maxim. | O | O |  |
| Asteraceae | *Cirsium japonicum*var.*maackii* (Regel) Kitam. | O | O |  |
| Asteraceae | *Conyza canadensis* (L.) Cronquist | O | X | Na |
| Asteraceae | *Conyza sumatrensis* (Retz.) E. Walker | O | O | Na |
| Asteraceae | *Crepidiastrum denticulatum* (Houtt.) Pak & Kawano | O | O |  |
| Asteraceae | *Crepidiastrum lanceolatum* (Houtt.) Nakai | O | X |  |
| Asteraceae | *Dendranthema boreale* (Makino) Ling | O | O |  |
| Asteraceae | *Dendranthema zawadskii*var.*latilobum* (Maxim.) Kitam. | O | O |  |
| Asteraceae | *Erigeron annuus* (L.) Pers. | X | O | Na |
| Asteraceae | *Eupatorium makinoi*var.*oppositifolium* (Koidz.) Kawah. & Yahara | O | O |  |
| Asteraceae | *Farfugium japonicum* (L.) Kitam. | O | O |  |
| Asteraceae | *Lactuca indica* L. | O | X |  |
| Asteraceae | *Ligularia stenocephala* (Maxim.) Matsum. & Koidz. | X | O |  |
| Asteraceae | *Pseudognaphalium affine* (D. Don) Anderb. | O | O |  |
| Asteraceae | *Saussurea gracilis* Maxim. | X | O |  |
| Asteraceae | *Saussurea polylepis* Nakai | X | O | Ed |
| Asteraceae | *Sigesbeckia pubescens* (Makino) Makino | O | X |  |
| Asteraceae | *Sonchus oleraceus* L. | O | X | Na |
| Asteraceae | *Youngia japonica*subsp.*elstonii* (Hochr.) Babc. & Stebbins | O | O |  |
| **Austrobaileyales** | | | | |
| Schisandraceae | *Kadsura japonica* (L.) Dunal | X | O |  |
| **Buxales** | | | | |
| Buxaceae | *Buxus microphylla*var.*insularis* Nakai | O | O |  |
| **Campanulales** | | | | |
| Campanulaceae | *Adenophora triphylla var. japonica* (Regel) H. Hara | O | O |  |
| Campanulaceae | *Codonopsis lanceolata* (Siebold & Zucc.) Trautv. | X | O |  |
| Campanulaceae | *Platycodon grandiflorum* (Jacq.) A. DC. | O | X |  |
| **Capparales** | | | | |
| Brassicaceae | *Brassica juncea* (L.) Czern. | O | X | Ar |
| Brassicaceae | *Brassica napus* L. | O | X | Ar |
| **Caryophyllales** | | | | |
| Aizoaceae | *Tetragonia tetragonoides* (Pall.) Kuntze | O | X |  |
| Amaranthaceae | *Achyranthes bidentata*var. *japonica* Miq. | O | O |  |
| Amaranthaceae | *Chenopodium album* L. | O | X | Na |
| Caryophyllaceae | *Cerastium glomeratum* Thuill. | O | X | Na |
| Caryophyllaceae | *Silene aprica*var. *oldhamiana* (Miq.) C. Y. Wu | O | O |  |
| Caryophyllaceae | *Stellaria aquatica* (L.) Scop. | O | O |  |
| Phytolaccaceae | *Phytolacca acinosa* Roxb. | O | O | Ar |
| Phytolaccaceae | *Phytolacca americana* L. | O | X | Na |
| Polygonaceae | *Persicaria lapathifolia* (L.) Delarbre | O | O |  |
| Polygonaceae | *Rumex crispus* L. | O | O | Na |
| Portulacaceae | *Portulaca oleracea* L. | O | X |  |
| **Celastrales** | | | | |
| Celastraceae | *Euonymus japonicus* Thunb. | O | O |  |
| **Crossosomatales** | | | | |
| Staphyleaceae | *Euscaphis japonica* (Thunb.) Kanitz | X | O |  |
| **Commelinales** | | | | |
| Commelinaceae | *Commelina communis* L. | O | O |  |
| **Cornales** | | | | |
| Aucubaceae | *Aucuba japonica* Thunb. | X | O |  |
| Cornaceae | *Cornus kousa* F. Buerger ex Miq. | X | O |  |
| **Cucurbitales** | | | | |
| Cucurbitaceae | *Gynostemma pentaphyllum* (Thunb.) Makino | X | O |  |
| **Dioscoreales** | | | | |
| Dioscoreaceae | *Dioscorea japonica* Thunb. | O | O |  |
| Dioscoreaceae | *Dioscorea quinquelobata* Thunb. | O | O |  |
| **Dipsacales** | | | | |
| Caprifoliaceae | *Lonicera japonica* Thunb. | O | O |  |
| Caprifoliaceae | *Patrinia villosa* (Thunb.) Juss. | O | O |  |
| Viburnaceae | *Viburnum carlesii* var. *bitchiuense* Thunb. | O | O |  |
| Viburnaceae | *Viburnum dilatatum* Thunb. | X | O |  |
| Viburnaceae | *Viburnum japonicum* (Thunb.) C. K. Spreng. | O | X |  |
| **Ebenales** | | | | |
| Styracaceae | *Styrax japonicus* Siebold & Zucc. | X | O |  |
| **Ericales** | | | | |
| Ericaceae | *Rhododendron mucronulatum* Turcz. | X | O |  |
| Pentaphylacaceae | *Eurya emarginata* (Thunb.) Makino | O | O |  |
| Pentaphylacaceae | *Eurya japonica* Thunb. | X | O |  |
| Primulaceae | *Ardisia japonica* (Thunb.) Blume | X | O |  |
| Primulaceae | *Lysimachia mauritiana* Lam. | O | X |  |
| Theaceae | *Camellia japonica* L. | O | O |  |
| **Fabales** | | | | |
| Fabaceae | *Lespedeza bicolor* Turcz. | O | O |  |
| Fabaceae | *Pueraria lobata* (Willd.) Ohwi | O | O |  |
| Fabaceae | *Rhynchosia volubilis* Lour. | O | X |  |
| Fabaceae | *Trifolium repens* L. | O | O | Na |
| **Fagales** | | | | |
| Betulaceae | *Carpinus turczaninowii* Hance | O | O |  |
| Fagaceae | *Castanopsis sieboldii* (Makino) Hatus. ex T. Yamaz. & Mashiba | O | O |  |
| Fagaceae | *Quercus acuta* Thunb. | X | O |  |
| **Gentianales** | | | | |
| Apocynaceae | *Trachelospermum asiaticum* (Siebold & Zucc.) Nakai | O | O |  |
| Apocynaceae | *Cynanchum wilfordii* (Maxim.) Hemsl. | O | X |  |
| Rubiaceae | *Galium spurium* L. | O | O |  |
| Rubiaceae | *Oldenlandia strigulosa* Bartl. ex DC. | O | X |  |
| Rubiaceae | *Paederia foetida* L. | O | O |  |
| Rubiaceae | *Rubia cordifolia* L. | O | O |  |
| **Lamiales** | | | | |
| Lamiaceae | *Callicarpa japonica*var. *luxurians* Rehder | O | O |  |
| Lamiaceae | *Clerodendrum trichotomum* Thunb. | O | O |  |
| Lamiaceae | *Isodon inflexus* (Thunb.) Kudô | O | O |  |
| Lamiaceae | *Scutellaria pekinensis* var. *transitra* (Makino) H. Hara | X | O |  |
| Oleaceae | *Fraxinus rhynchophylla* Hance | X | O |  |
| Oleaceae | *Fraxinus sieboldiana* Blume | X | O |  |
| Oleaceae | *Ligustrum japonicum* Thunb. | O | O |  |
| Oleaceae | *Ligustrum obtusifolium* Siebold & Zucc. | O | O |  |
| Phrymaceae | *Phryma leptostachya*var.*oblongifolia* (Koidz.) Honda | X | O |  |
| Plantaginaceae | *Plantago asiatica* L. | O | O |  |
| **Laurales** | | | | |
| Lauraceae | *Litsea japonica* (Thunb.) Juss. | O | O |  |
| Lauraceae | *Machilus thunbergii* Siebold & Zucc. | O | O |  |
| Lauraceae | *Neolitsea sericea* (Blume) Koidz. | O | O |  |
| **Liliales** | | | | |
| Liliaceae | *Lilium lancifolium* Thunb. | O | O |  |
| **Malpighiales** | | | | |
| Euphorbiaceae | *Mallotus japonicus* (L. f.) Müll. | O | O |  |
| Violaceae | *Viola* sp. | O | O |  |
| **Malvales** | | | | |
| Malvaceae | *Corchoropsis tomentosa* (Thunb.) Makino | O | O |  |
| Malvaceae | *Grewia parviflora* Bunge | O | O |  |
| **Oxalidales** | | | | |
| Oxalidaceae | *Oxalis corniculata* L. | O | O | Ar |
| **Poales** | | | | |
| Cyperaceae | *Carex ciliatomarginata* Nakai | X | O |  |
| Cyperaceae | *Carex breviculmis* R. Br. | O | O |  |
| Cyperaceae | *Carex lanceolata* Boott | O | O |  |
| Cyperaceae | *Carex wahuensis*var.*robusta* (Franch. & Sav.) Franch. & Sav. | O | O |  |
| Cyperaceae | *Fimbristylis sieboldii* Miq. ex Franch. & Sav. | O | X |  |
| Poaceae | *Arundinella hirta* (Thunb.) Tanaka | O | O |  |
| Poaceae | *Avena fatua* L. | O | X | Na |
| Poaceae | *Brachypodium sylvaticum* (Huds.) P. Beauv. | X | O |  |
| Poaceae | *Bromus catharticus* Vahl | O | X | Na |
| Poaceae | *Calamagrostis arundinacea* (L.) Roth | X | O |  |
| Poaceae | *Cleistogenes hackelii* (Honda) Honda | O | X |  |
| Poaceae | *Digitaria ciliaris* (Retz.) Koeler | O | X |  |
| Poaceae | *Elymus tsukushiensis* Honda | O | O |  |
| Poaceae | *Festuca arundinacea* Schreb. | O | O | Na |
| Poaceae | *Festuca ovina* L. | O | O |  |
| Poaceae | *Miscanthus sinensis* Andersson | O | O |  |
| Poaceae | *Oplismenus undulatifolius* (Ard.) Roem. & Schult. | O | O |  |
| Poaceae | *Phaenosperma globosa* Munro & Benth. | O | O |  |
| Poaceae | *Poa sphondylodes* Trin. | O | O |  |
| Poaceae | *Polypogon fugax* Nees ex Steud. | O | X |  |
| Poaceae | *Setaria viridis* (L.) P. Beauv. | O | O |  |
| **Ranunculales** | | | | |
| Lardizabalaceae | *Stauntonia hexaphylla* Decne. | X | O |  |
| Menispermaceae | *Cocculus trilobus* (Thunb.) DC. | O | O |  |
| Menispermaceae | *Sinomenium acutum* (Thunb.) Rehder & E. H. Wilson | O | X |  |
| Papaveraceae | *Corydalis platycarpa* (Maxim. ex Palib.) Makino | O | X |  |
| Ranunculaceae | *Clematis apiifolia* DC. | X | O |  |
| Ranunculaceae | *Clematis terniflora* DC. | X | O |  |
| Ranunculaceae | *Thalictrum uchiyamae* Nakai | O | O |  |
| **Rosales** | | | | |
| Cannabaceae | *Celtis sinensis* Pers. | O | O |  |
| Elaeagnaceae | *Elaeagnus glabra* Thunb. | O | X |  |
| Elaeagnaceae | *Elaeagnus macrophylla* Thunb. | O | O |  |
| Moraceae | *Broussonetia*×*kazinoki* Siebold ex Siebold & Zucc. | O | O |  |
| Moraceae | *Broussonetia papyrifera* (L.) L’Hér. ex Vent. | O | X |  |
| Moraceae | *Ficus erecta* Thunb. | O | O |  |
| Moraceae | *Ficus oxyphylla* Miq. ex Zoll. | O | O |  |
| Moraceae | *Morus bombycis* Koidz. | X | O |  |
| Rhamnaceae | *Rhamnus rugulosa* Hemsl. | X | O |  |
| Rhamnaceae | *Sageretia theezans* (Osbeck) M. C. Johnst. | X | O |  |
| Rosaceae | *Potentilla gageodoensis* M. Kim | O | O | Ed |
| Rosaceae | *Prunus sargentii* Rehder | X | O |  |
| Rosaceae | *Rhaphiolepis indica*var.*umbellata* (Thunb.) Ohashi | O | O |  |
| Rosaceae | *Rosa multiflora* Thunb. | O | O |  |
| Rosaceae | *Rubus coreanus* Miq. | O | O |  |
| Rosaceae | *Rubus crataegifolius* Bunge | O | O |  |
| Rosaceae | *Rubus hirsutus* Thunb. | O | O |  |
| Rosaceae | *Rubus parvifolius* L. | O | O |  |
| Urticaceae | *Boehmeria pannosa* Nakai & Satake ex Oka | O | O |  |
| Urticaceae | *Boehmeria nivea* (L.) Gaudich. | O | O |  |
| Urticaceae | *Pilea peploides* (Gaudich.) Hook. & Arn. | O | O |  |
| **Santalaes** | | | | |
| Viscaceae | *Korthalsella japonica* (Thunb.) Engl. | X | O |  |
| **Sapindales** | | | | |
| Rutaceae | *Zanthoxylum schinifolium* Siebold & Zucc. | X | O |  |
| Rutaceae | *Zanthoxylum ailanthoides* Siebold & Zucc. | O | O |  |
| Sapindaceae | *Acer pictum*var.*mono* (Maxim.) Franch. | O | X |  |
| Simaroubaceae | *Rhus javanica* L. | O | O |  |
| **Saxifragales** | | | | |
| Crassulaceae | *Hylotelephium spectabile* (Boreau) H. Ohba | O | O |  |
| Crassulaceae | *Meterostachys sikokianus* (Makino) Nakai | X | O |  |
| Crassulaceae | *Sedum bulbiferum* Makino | O | X |  |
| Crassulaceae | *Sedum kamtschaticum* Fisch. & C. A. Mey. | O | O |  |
| Crassulaceae | *Sedum oryzifolium* Makino | O | X |  |
| Crassulaceae | *Sedum polytrichoides* Hemsl. | O | O |  |
| Daphniphyllaceae | *Daphniphyllum macropodum* Miq. | O | O |  |
| **Solanales** | | | | |
| Solanaceae | *Solanum lyratum* Thunb. | O | O |  |
| Solanaceae | *Solanum nigrum* L. | O | X | Ar |
| **Vitales** | | | | |
| Vitaceae | *Ampelopsis brevipedunculata* (Maxim.) Trautv. | O | O |  |
| Vitaceae | *Parthenocissus tricuspidata* (Siebold & Zucc.) Planch. | O | O |  |
| Vitaceae | *Vitis ficifolia var. sinuata* (Regel) H. Hara | O | O |  |

^1^O: Presence, X: absence

^2^En: endangered species, Ed: Korea endemic species, Ar: Archaeophyte, Na: Naturalized species
